# Cardiometabolic Risk Factors and Domain-Specific Cognitive Function in Prediabetes and Type 2 Diabetes

**DOI:** 10.3390/medsci14050520

**Published:** 2026-08-26

**Authors:** Rohit Hariharan, Simon M. Bell, Arshad Majid, Jack Feehan, Barbora de Courten, Aya Mousa

**Affiliations:** 1 Monash Centre for Health Research and Implementation, Faculty of Medicine, Nursing and Health Sciences, Monash University, 43-51 Kanooka Grove, Clayton, VIC 3168, Australia; rohit.hariharan@monash.edu; 2 Sheffield Institute for Translational Neuroscience, School of Medicine and Population Health, University of Sheffield, 385a Glossop Rd., Broomhall, Sheffield S10 2HQ, UK; 3 NIHR Sheffield Biomedical Research Centre, University of Sheffield and Sheffield Teaching Hospitals NHS Foundation Trust, Glossop Rd, Broomhall, Sheffield S10 2JF, UK; 4 Neuroscience Institute, University of Sheffield, Firth Court, Sheffield S10 2TN, UK; 5 School of Health and Biomedical Sciences, STEM College, RMIT University, 30 Janefield Drive, Bundoora, VIC 3083, Australia

**Keywords:** prediabetes, type 2 diabetes, cardiometabolic risk, lipids, HbA1c, cognitive function

## Abstract

**Background**: Cardiometabolic risk factors are increasingly recognised as contributors to cognitive decline, yet their associations with domain-specific cognitive performance in adults with prediabetes or well-controlled type 2 diabetes (T2DM) remain poorly characterised. **Methods**: This cross-sectional study included 49 adults with prediabetes or well-controlled T2DM (managed by diet or metformin alone; HbA1c < 8%). Cognitive function was assessed using the Cambridge Neuropsychological Test Automated Battery (CANTAB), Trail Making Tests (TMTs) parts A and B, Stroop, and Digit Symbol Substitution Test (DSST). Multivariable linear regression examined associations between cardiometabolic predictors (adiposity [DXA], glycaemic indices [OGTT], and serum lipids) and cognitive outcomes, adjusting for age, BMI, and education. **Results**: Higher HbA1c was independently associated with increased false alarms on the rapid visual processing task (RVPPFA; β = 0.04 [95% CI 0.01, 0.07]; *p* = 0.015); fasting and 2 h glucose were not associated with any cognitive measure. Higher triglycerides were associated with lower sustained attention accuracy (RVPA; β = −0.02 [95% CI −0.04, −0.004]; *p* = 0.016) and lower DSST scores (β = −4.61 [95% CI −8.81, −0.40]; *p* = 0.033). Higher HDL cholesterol was independently associated with better sustained attention (RVPA; β = 0.08 [95% CI 0.02, 0.15]; *p* = 0.017). No consistent associations were found between adiposity measures and any cognitive outcome, with no effect modification by sex or diabetes status (all P*_interaction_* > 0.05). **Conclusions**: In cognitively healthy adults with prediabetes or well-controlled T2DM, higher HbA1c and triglycerides and lower HDL were associated with poorer domain-specific cognitive performance in the absence of overt cognitive impairment. Larger longitudinal studies are needed to establish whether these associations represent early cognitive changes and to determine whether reducing cardiometabolic risk can preserve cognitive function.

## 1. Introduction

Population ageing is increasingly recognised as a major driver of the global burden of disease. In 2020, approximately 727.6 million individuals were aged over 65 years, representing 9.33% of the global population [1]. The rate of population ageing is projected to accelerate, with the number of individuals aged ≥60 years expected to double to 2.1 billion by 2050, and those aged ≥80 years to triple between 2020 and 2050 [1,2]. In parallel, the prevalence of cardiometabolic disorders, including obesity, prediabetes, type 2 diabetes (T2DM), and dyslipidaemia, also increases with age. These conditions are now recognised risk factors for cognitive decline and dementia [3,4], with both obesity and diabetes among several modifiable risk factors estimated to contribute to 40% of dementia cases [5]. Impaired cerebral perfusion, insulin resistance, chronic inflammation, and oxidative stress are proposed mechanisms by which cardiometabolic risk contributes to declines in cognitive performance [6,7,8].

Cognitive impairment may manifest as global cognitive dysfunction or as deficits within one or more specific neuropsychological domains, such as memory, pattern recognition, attention, and executive function. Individuals with prediabetes, characterised by dysregulated glucose metabolism below the threshold for T2DM (i.e., fasting plasma glucose of 6.1 to 6.9 mmol/L or 2–h post-load plasma glucose of 7.8 to 11.0 mmol/L, or HbA1c of 5.7% to 6.4%) [9,10], have shown modest impairments in memory and executive function, which may worsen with diabetes progression or if left untreated [11,12]. T2DM is associated with a 19% higher risk of cognitive decline over a 20-year period [13], with ageing compounding this effect [14]. Beyond glucose metabolism, other cardiometabolic risk factors also influence cognitive performance. Obesity, reflected by an increased adiposity, waist-to-hip ratio, and/or body mass index (BMI), has been associated with poorer cognitive function [15]. Elevated triglycerides and/or an unfavourable lipid profile (i.e., dyslipidaemia) are common in T2DM [16,17], and have been implicated in neurodegeneration by promoting atherosclerosis and vascular damage of cerebral arteries [18].

Despite growing recognition of the relationship between cardiometabolic health and cognitive function, the associations between key cardiometabolic risk factors and domain-specific cognitive performance remain unclear, particularly among individuals with prediabetes or well-controlled T2DM who are otherwise cognitively healthy but may exhibit early mild impairments. This is important, as identifying cognitive impairment or dementia at the earliest possible point in the disease trajectory is critical to optimising treatment outcomes and can inform the development of novel metabolic biomarkers for these disease states. A comprehensive approach integrating both cardiometabolic and cognitive measures is therefore needed to characterise these relationships and provide insight into underlying pathophysiological pathways.

To address this, the present exploratory study aims to examine the relationships between body composition, glycaemic measures, and lipid profiles with cognitive performance among individuals with prediabetes or well-controlled T2DM who are otherwise considered cognitively healthy.

## 2. Materials and Methods

### 2.1. Participants

This study is an exploratory cross-sectional analysis of baseline data from a double-blind, randomised, placebo-controlled trial that examined the impact of 14 weeks of carnosine supplementation on cardiometabolic outcomes in individuals with prediabetes or type 2 diabetes. The detailed methodology for the trial is described in the published protocol [19], and the primary findings are reported elsewhere [20]. In brief, eligible participants were aged 18–70 years and were diagnosed with prediabetes or type 2 diabetes through an oral glucose tolerance test (OGTT) and HbA1c, following WHO 2019 criteria [21]. Only those managing their condition through diet and/or with metformin only were eligible for inclusion, and all participants were cognitively healthy based on self-reported history. Exclusion criteria included HbA1c levels exceeding 8% at screening; a BMI over 40 kg/m^2^; significant weight fluctuations (greater than 5 kg in the previous six months); smoking; excessive alcohol consumption (>4 standard drinks per week for men or >2 for women) [22]; renal failure (estimated glomerular filtration rate < 30 mL/min); active cancer within the previous five years; acute inflammation or infection; the use of anti-inflammatory medications or any medications or dietary supplements known to affect cardiometabolic or cognitive outcomes; and the presence of cardiovascular, endocrine, respiratory, haematological, gastrointestinal, or central nervous system disorders, including cognitive and psychiatric conditions. Participants with T2DM were required to have been on stable treatment for at least three months prior to enrolment and were advised not to change their treatment during the study. Pregnant or lactating women were also excluded.

Participants were recruited through advertisements in newspapers, radio, and posters. Prospective participants underwent an initial screening via a telephone-based questionnaire, and those meeting preliminary eligibility criteria were invited for an in-person screening visit at the Clinical Trials Centre within the Monash Health Translational Research Precinct in Melbourne, Australia.

### 2.2. Outcome Measures

At the screening visit, a medical review and a 75 g oral glucose tolerance test (OGTT) were performed after an overnight fast to obtain fasting and post-load blood glucose levels, as well as HbA1c, to confirm prediabetes or diabetes status based on WHO (2019) guidelines [21]. Additional fasting blood samples were collected and analysed at Monash Health Pathology (Melbourne, Australia), which comprised a standard biochemical panel (renal and liver function, full blood count) to exclude other underlying conditions and for measurement of serum lipids by commercial immunoassays to characterise cardiometabolic risks. Demographic characteristics including age and level of education (primary, secondary, tertiary or above) were collected via self-reported answers in the CANTAB application. Anthropometric measurements included the BMI, calculated from weight (kg)/height^2^ (m^2^); the waist-to-hip ratio, derived from the waist circumference/hip circumference when measured during the initial medical review; and the total body fat (%), measured by dual-energy X-ray absorptiometry (DXA).

Cognitive function was assessed using four cognitive instruments administered in both written and digital formats. These included the Cambridge Neuropsychological Test Automated Battery (CANTAB), the Trail Making Tests (A&B), and the Stroop test, which were performed using a digital tablet (iPad, iOS v.10, Apple Inc., Melbourne, Australia), as well as the Digit Symbol Substitution Test, which was administered on paper. Descriptions of these assessments and their relevant domains and score interpretations are described in Table 1. Assessments took place during working hours (9 a.m.–5 p.m.) in the same location and in the same sequence, although they were not conducted at the same time of day. Before starting each test, participants received a brief explanation of the procedure. To minimise external distractions, the tests were conducted in a quiet, well-lit, and controlled environment, with only the participant and researcher present. Participants were not required to fast before testing.

### 2.3. Ethics

All participants provided written informed consent prior to undergoing baseline screening procedures. The trial was registered on clinicaltrials.gov (NCT02917928; 28 September 2016) and carried out according to the published protocol [19] and Standard Protocol Items: Recommendations for Interventional Trials (SPIRIT) guidelines [23], and was reported in line with STROBE guidelines [24]. Ethical approval was granted by the Monash Health Human Research Ethics Committee (Ref: 16061A; 14 April 2016), and all aspects of the trial adhered to the principles of the Declaration of Helsinki [25].

### 2.4. Statistical Analysis

Statistical analyses were performed using JASP (v0.95.4; University of Amsterdam, Amsterdam, The Netherlands) and SPSS (v26, IBM Corp, Armonk, New York, NY, USA). Normality of cognitive variables was assessed through visual inspection of histograms, along with the Shapiro–Wilk and Kolmogorov–Smirnov tests to evaluate skewness and kurtosis. Descriptive statistics were presented as means with standard deviations (SDs) for normally distributed continuous data or as medians with interquartile ranges (IQRs) for non-normally distributed continuous data, and as frequencies and proportions for categorical data. For the CANTAB, key variables were analysed following the guidelines provided in the CANTAB handbook [26].

Simple and multivariable linear regression models were used to explore relationships between cardiometabolic parameters as predictors (anthropometric, glycaemic, and lipid measures) and cognitive scores as outcomes. Four separate models were fitted for each metabolic predictor; Model 1 (unadjusted); Model 2, adjusted for age; Model 3, adjusted for age and body mass index (BMI); and Model 4, adjusted for age, BMI, and level of education. Covariates were limited to age, BMI, and level of education given the sample size, as additional adjustment would risk model overfitting. Where significant associations were identified in these regression models, potential effect modification by sex and diabetes status was assessed by incorporating interaction terms into the fully adjusted models. Sensitivity analyses were performed with additional adjustment for metformin use to examine changes in the results. A two-sided *p*-value < 0.05 was considered statistically significant. To aid interpretation of effect magnitudes, standardised estimates were derived for significant associations in the fully adjusted models by multiplying each regression coefficient with the standard deviation of the cardiometabolic exposure and dividing by the SD of the corresponding cognitive outcome to express each association per one standard deviation of exposure in standard deviation units of the outcome. Across the fully adjusted models, 11 cardiometabolic predictors were examined in relation to 17 cognitive outcomes. Given the post hoc exploratory nature of the study, this secondary analysis was not powered for cognitive outcomes, and there were no corrections for multiple testing. Significant findings should therefore be interpreted as nominal and hypothesis-generating rather than confirmatory, requiring validation in larger adequately powered cohorts.

## 3. Results

### 3.1. Sample Characteristics

The demographic, anthropometric, clinical, biochemical, and cognitive characteristics of participants are presented in Table 2. Of the 50 recruited participants, one participant with normoglycaemia was excluded, leaving 49 for inclusion in the final analysis. Approximately two-thirds of the cohort were males (*n* = 33; 67%), with 23 participants (47%) classified as having T2DM and 26 (53%) with prediabetes per WHO (2019) criteria [21]. Nearly half of the participants were aged 50 years and over (47%; n = 23). The majority were overweight (BMI ≥ 25–29.9 kg/m^2^; 53%, n = 26) or obese (BMI ≥ 30 kg/m^2^; 39%, n = 19), with only four participants within the normal BMI range. Participants were considered cognitively healthy based on self-reports and medical histories.

### 3.2. Associations Between Cognitive and Cardiometabolic Measures

#### 3.2.1. Adiposity

The body fat percentage and waist-to-hip ratio were not associated with any cognitive measure across the CANTAB, Trail Making Tests (TMTs), Stroop, or Digit Symbol Substitution Test (DSST) in unadjusted or multivariable models (Table 3, Table 4 and Table 5). BMI was similarly not associated with any cognitive outcome except TMT-B’s completion time, where a higher BMI was associated with faster completion in the fully adjusted model (β = −2.51 [95% CI −4.76, −0.25]; *p* = 0.03) but not in unadjusted or partially adjusted models (Table 4). Sensitivity analyses with further adjustment for metformin use did not alter these findings, and no significant interaction effects with sex or diabetes status were observed (*P_interaction_* > 0.08; Table 4).

#### 3.2.2. Glycaemic Measures

Higher HbA1c was associated with a greater probability of false alarms on the CANTAB rapid visual processing task (RVPPFA) in the unadjusted model (β = 0.03 [95% CI 0.003, 0.07]; *p* = 0.03) (Table 3). This association remained significant after adjusting for age (*p* = 0.011), BMI (*p* = 0.014), and level of education (β = 0.04 [95% CI 0.01, 0.07]; *p* = 0.015). No significant interaction effects with sex or diabetes status were observed for this association (*P_interaction_* >0.26; Table 3). Fasting and 2–h post-load glucose were not associated with any cognitive measures in univariable or multivariable analyses (Table 3, Table 4 and Table 5; Supplemental Table S1). Additional adjustment for metformin use did not alter results for any of these glycaemic parameters.

#### 3.2.3. Lipid Profile

Higher triglyceride concentrations were associated with lower sustained attention accuracy on the CANTAB rapid visual processing task (RVP A prime [RVPA]; β = −0.02 [95% CI −0.04, −0.01]; *p* = 0.008; Table 3). This remained significant after adjustment for age (*p* = 0.012), BMI (*p* = 0.014), and level of education (β = −0.02 [95% CI −0.04, −0.004]; *p* = 0.016), with no effect modification by sex or diabetes status (Table 3). Higher triglycerides were also associated with slower processing speed as measured by RVP median latency (RVPMDL) in age-adjusted and BMI-adjusted models (β = 27.19 [95% CI 1.86, 52.52]; *p* = 0.036), although they were attenuated in the fully adjusted model (*p* = 0.057). There was a significant interaction with diabetes status for this association (β = 74.10 [95% CI 24.55, 123.66]; *P_interaction_* = 0.004; Table 3).

In addition to CANTAB domains, higher triglycerides were associated with a higher TMT B/A ratio in the unadjusted (β = 0.42 [95% CI 0.01, 0.83]; *p* = 0.045), age-adjusted (*p* = 0.045), and age- and BMI-adjusted models (*p* = 0.043), but this did not reach statistical significance in the fully adjusted model (*p* = 0.050), and there were no interactions with sex or diabetes status (*P_interaction_* > 0.42; Table 4). Higher triglycerides were also associated with lower DSST scores across all adjusted models (all *p* ≤ 0.03), including in the fully adjusted model (β = −4.61 [95% CI −8.81, −0.40]; *p* = 0.033), with no interactions with sex or diabetes status (*P_interaction_* > 0.31; Table 5).

Higher concentrations of high-density lipoprotein cholesterol (HDL-C) were associated with better sustained attention (RVPA; β = 0.08 [95% CI 0.02, 0.15]; *p* = 0.010) in CANTAB, which remained significant across all multivariable models (all *p* < 0.02), with no significant interactions with sex or diabetes status (*P_interaction_* > 0.54; Table 3). Other lipid measures, including total cholesterol, low-density lipoprotein cholesterol (LDL-C), and LDL:HDL ratio, were not associated with any cognitive measures in both univariable and multivariable analyses (Table 3, Table 4 and Table 5; Supplemental Table S1). The results for all lipid measures were not altered in sensitivity analyses with additional adjustment for metformin use.

To aid interpretation, the effect magnitudes of significant associations are presented in Table 6, expressed as per 1 SD of cardiometabolic exposure relative to 1 SD of the cognitive outcome. All associations ranged between −0.36 SD and + 0.4 SD (Table 6).

## 4. Discussion

This study explored associations between cardiometabolic risk factors and domain-specific neurocognitive performance in cognitively healthy adults with prediabetes or well-controlled T2DM. Our findings suggest that higher HbA1c was associated with a higher false alarm rate on a sustained attention task, and that lipid dysregulation, particularly higher triglycerides and lower HDL-C concentrations, was associated with poorer sustained attention, and processing speed. These associations were independent of age, BMI, and education. Notably, these were observed in the absence of overt cognitive impairment and under relatively well-controlled metabolic conditions, raising the possibility that subtle cognitive differences may be detectable early in cardiometabolic disease using targeted neuropsychological assessments. Effect magnitudes were modest (−0.36 SD to +0.41 SD), suggesting variations within normal cognitive performance, rather than clinically significant cognitive impairment.

Adiposity measures, including body fat percentage, waist-to-hip ratio, and BMI, were not consistently associated with cognitive outcomes in this study. The literature on adiposity and cognition is inconsistent. Some studies have reported no associations between BMI and cognitive measures in cognitively healthy older adults [27], while others have reported detrimental effects on brain volumes in T2DM [28]. Sex-specific associations have also been reported [29,30] but could not be explored here given the unequal sex distribution and modest sample size. Interestingly, Xing et al. [31] found that BMI was not associated with DSST or Stroop performance in T2DM, consistent with our findings, though unexpected positive associations with memory scores were observed. These echo similarly counterintuitive reports that moderately higher BMI may protect against grey matter loss [32] and cognitive decline [33] in later life. In our study, the isolated association between a higher BMI and faster TMT-B completion, present only in the fully adjusted model, may reflect the same paradox, though its model dependency and small sample size warrant cautious interpretation. Collectively, these findings suggest that the adiposity–cognition relationship is complex and requires further investigation in adequately powered studies with balanced sex representation.

Higher HbA1c was associated with increased false alarm responses on the Rapid Visual Processing task (RVPPFA), reflecting poorer inhibitory control, independent of age, BMI, and education. This is broadly consistent with evidence that chronic glycaemic exposure in prediabetes or T2DM is associated with cognitive decline [34,35,36,37], particularly in attention and executive function. However, prior studies largely relied on global composite scores or brief screening instruments and typically included individuals with more severe disease on complex medication regimens, introducing confounding by indication [38,39]. Here, these associations were detectable in a cognitively healthy cohort managed with diet or metformin alone, with HbA1c below 8%, indicating that this association was observable at lower levels of glycaemic exposure than have typically been demonstrated in prior work. This points toward glycaemia-related attentional dysregulation potentially emerging earlier than previously recognised, an intriguing hypothesis that warrants formal testing. The specificity to inhibitory control is also of interest; false alarm responses reflect frontoparietal network function [40], suggesting that this network may be particularly vulnerable to early glycaemic dysregulation. Notably, fasting and 2 h post-load glucose were not associated with any cognitive measure, while HbA1c remained significant across all models, pointing to a cumulative rather than acute glycaemic burden as the more relevant exposure. Indeed, glycaemic variability has been shown to damage neurons through glucose transporter mismatches [41], a cumulative process integrated over time by HbA1c but not captured by isolated glucose measurements, consistent with our findings.

Other mechanisms linking chronic hyperglycaemia and cognitive dysfunction include oxidative stress, endothelial dysfunction, and reduced cerebral perfusion, which directly contribute to neuronal injury [4,39]. Accumulation of advanced glycation end-products (AGEs), which bind to their receptor (RAGE), further perpetuates oxidative and cellular damage associated with up to three-fold increased odds of mild cognitive impairment (MCI) [42,43,44,45]. In parallel, cerebral insulin resistance impairs long-term potentiation of synaptic plasticity, a key process in memory formation and learning [8], by reducing long-term potentiation and brain-derived neurotrophic factor (BDNF) production [46,47]. Hyperglycaemia may also promote amyloid pathology through increased amyloid precursor protein expression [48]. While these mechanisms offer plausible explanations, the key pathways in this population require further investigation, ideally using combined neuroimaging, cognitive testing and biomarker-based approaches.

Lipid dysregulation was the most prominent cardiometabolic risk factor associated with cognitive performance in this study. Independent of age, BMI, and education, higher triglycerides were associated with lower accuracy on the sustained attention task (RVPA) and poorer processing speed (DSST) across all adjusted models, whereas higher HDL-C was associated with better sustained attention across all models. These findings are broadly consistent with prior evidence linking higher triglycerides to poorer executive function [49] and higher HDL-C to better cognitive performance in older adults [50]. Studies in T2DM cohorts have been mixed; some have reported associations between the triglyceride–glucose index with MCI, or between HDL-C and attention and memory [51], while others have reported no independent lipid–MCI associations [52,53] or report associations only for total and LDL-C [54]. These inconsistencies likely reflect differences in T2DM severity and medication use (e.g., statins), and a common reliance on global cognitive assessment tools such as the Montreal Cognitive Assessment (MoCA) [51,54], which may lack sensitivity to detect domain-specific associations. Here, we extend the literature by demonstrating that triglyceride and HDL-C associations with sustained attention, and processing speed may be detectable in a well-controlled, early-stage cardiometabolic cohort—a population largely overlooked in prior work—though these findings remain exploratory and require replication in future studies. Mechanistically, our findings mirror the contrasting roles of triglycerides and HDL-C in neurovascular health: triglyceride-rich lipoproteins promote blood–brain barrier dysfunction, endothelial injury, and amyloid dysmetabolism [55], whereas HDL-C exerts anti-inflammatory, antioxidative, and endothelial-protective effects that support cerebrovascular integrity [56]. These processes may impair or preserve cerebral perfusion and neural efficiency within frontoparietal networks underlying attention and processing speed [40], providing a neurovascular basis for the domain-specific associations observed here. These relationships may be further modulated by genetic susceptibility; apolipoprotein E4 (ApoE4), the strongest genetic risk factor for sporadic Alzheimer’s disease [57], regulates lipid transport within the central nervous system and has been linked to impaired lipid metabolism, neuroinflammation, and cognitive deficits across multiple domains [57,58]. The precise lipid-driven neurovascular mechanisms underlying cognitive risk, and their interactions with genetic factors, if any, await further study.

A key strength of this study is the use of a comprehensive cognitive assessment battery that combines domain-specific CANTAB measures with traditional neuropsychological tests (Stroop, TMTs, and DSST). This enabled the detection of subtle cognitive differences unlikely to be captured by global screening tools. Cardiometabolic risk was characterised using validated measures, including DXA-derived body composition, OGTT-based glycaemic measures and a full lipid panel. The inclusion of key covariates, including age, BMI, and education, strengthened the analyses, with education in particular as an important but less consistently accounted for factor in prior studies. Restricting inclusion to individuals managed by diet or metformin alone minimised confounding by disease severity and eligibility criteria of the parent trial excluded cardiovascular, renal, psychiatric, and central nervous system disease, as well as any medication or supplement affecting cardiometabolic or cognitive outcomes; therefore, no participant was taking antihypertensive or lipid-lowering medications. Blood pressure and depressive symptoms in this cohort were within normal ranges as reported previously [59,60], allowing early cardiometabolic–cognitive associations to be delineated more clearly than in previous studies of populations with more advanced disease.

Limitations of the study include the modest sample size, which restricted statistical power and precluded subgroup analyses, whereby sex and diabetes status could only be examined as interactions for exploratory purposes. With no correction for multiple testing and, given the large number of cognitive outcomes tested, there is an increased susceptibility for type I error; thus, individual findings should be interpreted with caution. The cross-sectional study design precluded causal inference and directionality, and reverse causation cannot be excluded. Although key confounders known to influence cognition (i.e., sex, level of education, and diabetes status) were incorporated in the analysis, the modest sample size precluded adjustment for a wider range of variables due to risk of overfitting. Thus, residual confounding by cardiometabolic (e.g., biomarkers), lifestyle (e.g., diet, sleep) and demographic factors (e.g., ethnicity categories) cannot be ruled out. Data on additional variables, including socioeconomic status or diabetes duration, were not measured in the parent trial, and a healthy control group was not included; aspects which should be considered in future studies. Cognitive health was established on the basis of self-reports and medical histories rather than cognitive screening, and could be subject to misclassification errors. The ApoE genotype was not assessed and may represent an unmeasured confounder or effect modifier, given its established role in lipid metabolism and cognitive decline. Finally, the cohort was predominantly male and highly selected, with strict inclusion criteria (HbA1c < 8%, well-controlled T2DM with diet or metformin only, BMI < 40 kg/m^2^ and absence of comorbidities). While the cohort’s cognitively healthy and metabolically well-controlled profile strengthens internal validity, it limits generalisability to other populations, including more diverse female populations, and those with more advanced or poorly controlled disease, complex polypharmacy, or established cognitive impairment. Given these constraints, the present findings should be considered hypothesis-generating rather than confirmatory, and are intended to inform the design of larger adequately powered longitudinal studies.

## 5. Conclusions

In cognitively healthy adults with prediabetes or well-controlled T2DM, glycaemic dysregulation and dyslipidaemia were associated with poorer cognitive performance, independent of age, BMI, and education, and in the absence of overt cognitive impairment. Larger prospective studies are needed to confirm whether these associations reflect early cognitive changes and whether optimising cardiometabolic risk factors can preserve cognitive function in this population.

## Figures and Tables

**Table 1:** Description of cognitive test procedures and interpretation of scores.

Measure	Description	Subcategories and Scoring	Score Indicator
**Cambridge Neuropsychological Test Automated Battery (CANTAB)**			
Delayed Match to Sample (DMS)	Evaluated attention and short-term visual recognition memory. Participants briefly viewed a complex pattern before it was obscured. After a delay (0, 4, or 12 s), they identified the correct pattern from a set of visually similar options. DMSPC: Delayed Match to Sample Percent Correct (simultaneous). DMSPCAD: Delayed Match to Sample Percent Correct (all delays).	DMSPC: Higher scores indicate better cognitive performance	⊕
		DMSPCAD: Higher scores indicate better cognitive performance	⊕
Paired Associates Learning (PAL)	Assessed visual episodic memory through a multi-stage process. Participants were shown six boxes, each containing a unique pattern, which were initially concealed. They were required to recall the original location of a displayed pattern. Errors resulted in a test reset until the error threshold was reached.	PALTEA: Lower scores indicate better cognitive performance	⊖
Reaction Time Index (RTI)	Evaluated processing and psychomotor speed. Participants were required to press and hold a button at the bottom of the screen. A green dot appeared in one of five circles in a random sequence, prompting participants to release the button, tap the dot, and return to the starting position. Reaction times were recorded in seconds, and includes the following: RTIFMDRT: Reaction Time Index Median Five Choice Reaction Time. RTIFMDMT: Reaction Time Index Median Five Choice Movement Time.	RTIFMDRT: Lower scores indicate better cognitive performance	⊖
		RTIFMDMT: Lower scores indicate better cognitive performance	⊖
Rapid Visual Processing (RVP)	Tested sustained attention by displaying a sequence of numbers in a central white box. Participants were required to press a button when target sequences appeared. The application calculated sensitivity to target detection and specificity by analysing false alarms and correct rejections. RVPA: Rapid Visual Processing A Prime RVPMDL: Rapid Visual Processing Median Latency RVPPFA: Rapid Visual Processing Probability of False Alarm	RVPA: Higher scores indicate better cognitive performance	⊕
		RVPMDL: Lower scores indicate better cognitive performance	⊖
		RVPPFA: Lower scores indicate better cognitive performance	⊖
Spatial Working Memory (SWM)	Assessed working memory and strategy. Participants searched for a hidden blue token among eight onscreen boxes, ensuring they did not revisit boxes where tokens had already been found. Difficulty increased with more tokens to locate. Performance was measured by the number of revisits to previously searched boxes and the consistency of strategies used across trials. SWMBE: Spatial Working Memory Between Errors. SWMS: Spatial Working Memory Strategy.	SWM: Lower scores indicate better cognitive performance	⊖
Motor Organisation Task (MOT)	Evaluated psychomotor function, motor control, and basic sensorimotor processing prior to completion of the main CANTAB. Participants were required to touch a series of crosses presented sequentially in different locations on the screen using the forefinger of their dominant hand. It was not intended as a primary cognitive outcome measure; the task was designed to familiarise participants with the touchscreen interface and assess visual comprehension, movement accuracy, and psychomotor responsiveness. Performance was assessed based on the speed and accuracy of responses. A lack of group differences on the MOT suggested that performance on subsequent cognitive tasks was less likely to be influenced by general motor impairment, low level perceptual deficits, or difficulty engaging with the test platform. MOTML: Motor organisation task mean latency.	MOTML: Lower scores indicate better cognitive performance	⊖
**Trail Making Tests (TMTs)**			
Trail Making Tests (TMT)	The Trail Making Tests (parts A and B) were administered using the “Trail Making Test UK/XIF5/1014/0071” application developed by Norgine Pharmaceuticals Ltd. The tests assessed processing speed, visual scanning, and sustained attention. In part A, participants used the index finger of their dominant hand to connect numbers 1 through 15, presented randomly in circles on an iPad screen. They were required to trace the sequence continuously without lifting their finger once the test began. In part B, the task was extended to an alternating sequence of numbers and letters (e.g., 1–A, 2–B, 3–C, etc.). Errors were immediately corrected by the test administrator before participants proceeded to the next step, ensuring only one attempt per test. The application measured the time taken to complete each section in seconds.	TMT-A, TMT-B and TMT B/A: Lower scores indicate better cognitive performance	⊖
**Stroop**			
Stroop	The Stroop test was administered using the Encephalapp application (https://www.encephalapp.com/). The Stroop test assesses executive function, selective attention, and inhibitory control. Participants completed 14 alternating trials involving two conditions. In the Stroop “off” condition, sequences of “####” symbols appeared and disappeared in three colours (red, blue, and green) at different screen locations. In the Stroop “on” condition, the words “red,” “green,” and “blue” were displayed in colours that did not match the textual meaning. Participants were required to select the correct colour by pressing corresponding buttons at the bottom of the screen. These response buttons were frequently rearranged in order and colour, necessitating attention to colour rather than word meaning. Any errors resulted in the participant restarting the specific trial. The application recorded the response times for each trial and provided an overall assessment of the completion time for both test conditions.	Offtime: Lower scores indicate better cognitive performance	⊖
		Ontime: Lower scores indicate better cognitive performance	⊖
**Digit Symbol Substitution Test (DSST)**			
Digit Symbol Substitution Test (DSST)	The Digit Symbol Substitution Test was conducted using a standardised questionnaire. It primarily assesses processing speed, attention, and concentration. Instructions provided at the top of the questionnaire displayed numbers ranging from 1 to 9, each paired with a corresponding non-linguistic symbol. Participants were required to complete randomly assigned numbered boxes by inputting the associated symbols. Initially, participants were given seven practice boxes to familiarise themselves with the task. Following this, a two-minute countdown commenced. Participants were instructed to focus and complete as many entries as possible within the allotted time, with a maximum achievable score of 93. The test administrator evaluated responses, awarding one point for each correct symbol placement, with 93 being the highest possible score.	DSST: Higher scores indicate better cognitive performance	⊕

⊕ = higher scores indicate better cognitive performance; ⊖ = lower scores indicate better cognitive performance.

**Table 2:** Participant characteristics overall and by diabetes status.

Variable	All Participants		Prediabetes (n = 26)		Diabetes (n = 23)	
	n	Mean ± SD or Median (IQR)	n	Mean ± SD or Median (IQR)	n	Mean ± SD or Median (IQR)
Demographics						
Age (years)	49	50.29 ± 10.70	26	48.88 ± 11.68	23	51.87 ± 9.47
Males, n (%)	49	33 (67%)	26	16 (62%)	23	17 (74%)
Metformin use, n (%)	49	18 (37%)	26	7 (27%)	23	11 (48%)
Anthropometry						
Total body fat, DXA (%)	49	34.74 (10.63)	26	36.42 (11.62)	23	32.62 (10.72)
BMI (kg/m^2^)	49	29.33 ± 4.08	26	30.06 ± 4.08	23	28.50 ± 4.00
Weight (kg)	49	86.52 ± 19.40	26	88.50 ± 18.64	23	84.27 ± 20.40
Height (cm)	49	170.81 ± 10.60	26	171.02 ± 9.77	23	170.58 ± 11.68
Waist circumference (cm)	47	101.31 ± 11.43	26	102.21 ± 12.48	21	100.19 ± 10.18
Hip circumference (cm)	46	104.56 ± 11.52	25	106.30 ± 13.44	21	102.43 ± 8.42
Waist-to-hip ratio	46	0.99 (0.09)	25	0.98 (0.11)	21	0.99 (0.05)
Glycaemic measures						
Fasting glucose (mmol/L)	49	6.10 (1.45)	26	5.83 (0.68)	23	7.03 (1.79)
2–h post-load glucose (mmol/L)	49	10.90 (4.85)	26	9.48 (2.06)	23	14.50 (4.20)
Haemoglobin A1c (%)	49	6.57 ± 0.81	26	6.17 ± 0.55	23	7.02 ± 0.82
Lipid profile						
Cholesterol (mmol/L)	49	5.40 ± 1.00	26	5.57 ± 1.03	23	5.2 ± 0.95
Triglycerides (mmol/L)	49	1.50 (1.30)	26	1.50 (0.50)	23	1.70 (1.70)
HDL cholesterol (mmol/L)	48	1.19 ± 0.26	25	1.21 ± 0.25	23	1.16 ± 0.27
LDL cholesterol (mmol/L)	47	3.36 ± 1.00	24	3.59 ± 0.93	23	3.12 ± 0.81
LDL/HDL ratio	47	2.93 ± 0.80	24	3.05 ± 0.82	23	2.79 ± 0.78
Digit Symbol Substitution Test						
Score (number of correct responses)	47	69.32 ± 15.39	25	70.32 ± 15.73	22	68.18 ± 15.28
Stroop tests						
Offtime (seconds)	47	61.81 ± 17.25	26	61.58 ± 15.64	21	62.10 ± 19.46
Ontime (seconds)	47	79.94 ± 14.78	26	79.94 ± 13.25	21	79.94 ± 16.82
Trail Making Tests						
Trail Making Test-A (seconds)	47	17.16 ± 5.32	26	16.86 ± 5.38	21	17.52 ± 5.36
Trail Making Test-B (seconds)	47	49.60 (35.50)	26	46.75 (35.10)	21	50.30 (31.8)
Trail Making Test B/A ratio	47	3.12 (1.72)	26	3.45 (1.65)	21	3.04 (1.86)
Cambridge Neuropsychological Test Automated Battery						
Delayed Match to Sample (DMS)						
DMS Percent Correct Simultaneous (%)	45	100.00 (0.00)	24	100.00 (10.00)	21	100.00 (0.00)
DMS Percent Correct All Delays (%)	45	87.00 (13.00)	24	87.00 (7.50)	21	80.00 (7.00)
Paired Associates Learning (PAL)						
PAL Total Errors Adjusted	45	15.00 (15.00)	24	14.5 (20.50)	21	15.00 (12.00)
Reaction Time Index (RTI)						
RTI Median Five Choice Movement Time (ms)	48	252.75 (113.00)	26	252.75 (134.50)	22	251.25 (93.50)
RTI Median Five Choice Reaction Time (ms)	48	399.60 ± 43.92	26	407.73 ± 44.45	22	390.00 ± 42.28
Rapid Visual Processing (RVP)						
RVP A-prime	45	0.90 (0.08)	24	0.90 (0.06)	21	0.90 (0.09)
RVP Median Response Latency	45	460.00 (89.00)	24	447.50 (70.25)	21	496.50 (86.00)
RVP Probability of False Alarms	45	0.006 (0.01)	24	0.006 (0.01)	21	0.008 (0.02)
Spatial Working Memory (SWM)						
SWM Between Errors	46	11.50 (13)	24	10.50 (15.00)	22	12.50 (14.00)
SWM Strategy	46	8.00 (5.00)	24	7.00 (5.00)	22	8.50 (4.00)
Motor Organisation Task (MOT)						
MOT Median Latency (ms)	48	827.60 ± 182.82	26	787.48 ± 181.91	22	875.02 ± 176.24

Data are presented as the mean ± standard deviation for normally distributed variables or median (IQR) for non-normally distributed variables, unless otherwise indicated. Abbreviations: BMI, body mass index; DXA, dual X-ray absorptiometry; HDL/LDL, high/low-density lipoprotein cholesterol; ms, milliseconds.

**Table 3:** Univariable and multivariable associations between cardiometabolic variables and selected CANTAB domains.

Predictor	Model	RTI Five Choice Median Movement Time (⊖)	*p*	RTI Five Choice Median Reaction Time (⊖)	*p*	RVP A-Prime (⊕)	*p*	RVP Median Latency (⊖)	*p*	RVP Probability of False Alarms (⊖)	*p*
		β (95% CI)		β (95% CI)		β (95% CI)		β (95% CI)		β (95% CI)	
Body mass index (kg/m^2^)	Unadjusted	0.71 (−6.01, 7.43)	0.83	0.16 (−3.25, 3.56)	0.93	−0.0003 (−0.01, 0.004)	0.91	−1.60 (−9.07, 5.88)	0.67	−0.0005 (−0.01, 0.01)	0.88
	+age	2.48 (−4.08, 9.03)	0.45	0.11 (−3.42, 3.64)	0.95	0.0004 (−0.004, 0.01)	0.88	0.44 (−6.93, 7.81)	0.91	−0.002 (−0.01, 0.01)	0.57
	+education	2.75 (−4.29, 9.79)	0.44	0.86 (−2.87, 4.60)	0.64	0.001 (−0.004, 0.01)	0.80	−2.17 (−9.59, 5.25)	0.56	−0.002 (−0.01, 0.01)	0.57
Waist-to-hip ratio	Unadjusted	5.13 (−288.15, 298.42)	0.97	−77.87 (−225.44, 69.71)	0.29	−0.04 (−0.24, 0.17)	0.72	−110.88 (−437.22, 215.45)	0.50	−0.04 (−0.31, 0.24)	0.78
	+age	−65.59 (−351.60, 220.42)	0.65	−78.98 (−231.75, 73.78)	0.30	−0.06 (−0.27, 0.14)	0.55	−190.59 (−504.76, 123.57)	0.23	0.01 (−0.27, 0.28)	0.96
	+education	−66.80 (−361.22, 227.61)	0.65	−65.03 (−220.50, 90.45)	0.40	−0.06 (−0.27, 0.15)	0.57	−244.31 (−545.09, 56.47)	0.11	0.01 (−0.28, 0.29)	0.95
Body fat (%)	Unadjusted	1.31 (−2.36, 4.99)	0.48	0.28 (−1.59, 2.15)	0.77	0.0002 (−0.002, 0.003)	0.90	0.48 (−3.60, 4.56)	0.81	−0.0002 (−0.004, 0.003)	0.89
	+age	1.90 (−1.62, 5.42)	0.28	0.27 (−1.64, 2.17)	0.78	0.0004 (−0.002, 0.003)	0.78	1.13 (−2.79, 5.05)	0.56	−0.001 (−0.004, 0.003)	0.71
	+education	1.90 (−1.66, 5.47)	0.29	0.23 (−1.67, 2.14)	0.81	0.0004 (−0.002, 0.003)	0.77	0.97 (−2.80, 4.73)	0.61	−0.001 (−0.004, 0.003)	0.71
Fasting glucose (mmol/L)	Unadjusted	−6.75 (−27.18, 13.69)	0.51	0.09 (−10.29, 10.47)	0.99	0.01 (−0.01, 0.02)	0.45	2.80 (−23.37, 28.97)	0.83	0.01 (−0.01, 0.04)	0.19
	+age	−10.14 (−29.73, 9.44)	0.30	0.20 (−10.41, 10.80)	0.97	0.01 (−0.01, 0.02)	0.56	−1.92 (−27.23, 23.39)	0.88	0.02 (−0.004, 0.04)	0.10
	+BMI	−10.72 (−30.43, 8.99)	0.28	0.17 (−10.59, 10.94)	0.97	0.01 (−0.01, 0.02)	0.56	−1.85 (−27.51, 23.81)	0.89	0.02 (−0.004, 0.04)	0.11
	+education	−10.62 (−30.83, 9.58)	0.30	1.21 (−9.64, 12.05)	0.82	0.01 (−0.01, 0.02)	0.53	−6.24 (−31.00, 18.52)	0.61	0.02 (−0.004, 0.04)	0.12
Two-hour post-load glucose (mmol/L)	Unadjusted	−4.39 (−12.04, 3.27)	0.26	−0.28 (−4.20, 3.65)	0.89	0.001 (−0.01, 0.01)	0.80	4.69 (−4.08, 13.45)	0.29	0.004 (−0.003, 0.01)	0.25
	+age	−6.22 (−13.55, 1.10)	0.09	−0.23 (−4.28, 3.81)	0.91	0.00 (−0.01, 0.01)	0.95	2.99 (−5.58, 11.56)	0.49	0.01 (−0.002, 0.01)	0.13
	+BMI	−5.88 (−13.49, 1.73)	0.13	−0.22 (−4.43, 3.99)	0.92	0.00 (−0.01, 0.01)	0.92	3.40 (−5.63, 12.43)	0.45	0.01 (−0.002, 0.01)	0.16
	+education	−5.91 (−13.61, 1.79)	0.13	−0.30 (−4.49, 3.90)	0.89	0.00 (−0.01, 0.01)	0.90	2.94 (−5.71, 11.59)	0.50	0.01 (−0.002, 0.01)	0.17
HbA1c (%)	Unadjusted	−2.37 (−34.39, 29.66)	0.88	−9.57 (−25.52, 6.38)	0.23	−0.001 (−0.02, 0.02)	0.95	2.58 (−36.05, 41.21)	0.89	0.03 (0.003, 0.07)	**0.030**
	+age	−7.36 (−38.20, 23.48)	0.63	−9.59 (−25.88, 6.70)	0.24	−0.003 (−0.03, 0.02)	0.82	−4.23 (−41.54, 33.08)	0.82	0.04 (0.01, 0.07)	**0.011**
	+BMI	−7.31 (−38.32, 23.70)	0.64	−9.59 (−26.07, 6.90)	0.25	−0.003 (−0.03, 0.02)	0.83	−4.04 (−42.03, 33.95)	0.83	0.04 (0.01, 0.07)	**0.014**
	+education	−7.00 (−38.58, 24.57)	0.66	−8.60 (−25.17, 7.97)	0.30	−0.002 (−0.03, 0.02)	0.86	−9.07 (−45.55, 27.41)	0.62	0.04 (0.01, 0.07)	**0.015**
	* sex	-	-	-	-	-	-	-	-	0.03 (−0.05, 0.11)	0.49
	* diabetes status	-	-	-	-	-	-	-	-	0.04 (−0.04, 0.12)	0.27
Total cholesterol (mmol/L)	Unadjusted	8.89 (−16.51, 34.28)	0.49	10.08 (−2.48, 22.64)	0.11	0.01 (−0.01, 0.02)	0.53	15.18 (−12.50, 42.87)	0.28	−0.003 (−0.03, 0.02)	0.80
	+age	3.47 (−21.41, 28.34)	0.78	10.68 (−2.25, 23.61)	0.10	0.004 (−0.01, 0.02)	0.68	9.46 (−17.71, 36.63)	0.49	0.001 (−0.02, 0.02)	0.95
	+BMI	4.26 (−20.82, 29.34)	0.73	10.78 (−2.34, 23.91)	0.11	0.004 (−0.01, 0.02)	0.67	9.70 (−17.93, 37.33)	0.48	0.0001 (−0.02, 0.02)	0.99
	+education	4.96 (−20.85, 30.78)	0.70	12.69 (−0.45, 25.84)	0.058	0.004 (−0.01, 0.02)	0.64	5.86 (−20.89, 32.61)	0.66	0.00005 (−0.02, 0.02)	1.00
HDL cholesterol (mmol/L)	Unadjusted	29.01 (−64.54, 122.56)	0.54	21.25 (−28.87, 71.36)	0.40	0.08 (0.02, 0.15)	**0.010**	−6.70 (−115.51, 102.10)	0.90	−0.002 (−0.09, 0.09)	0.97
	+age	8.99 (−82.20, 100.17)	0.84	23.02 (−28.59, 74.63)	0.37	0.08 (0.02, 0.14)	**0.017**	−31.50 (−136.95, 73.96)	0.55	0.01 (−0.08, 0.10)	0.77
	+BMI	13.62 (−79.26, 106.49)	0.77	23.51 (−29.32, 76.34)	0.38	0.08 (0.02, 0.15)	**0.016**	−31.22 (−139.08, 76.64)	0.56	0.01 (−0.08, 0.10)	0.83
	+education	14.03 (−78.20, 106.26)	0.76	23.82 (−27.81, 75.45)	0.36	0.08 (0.02, 0.15)	**0.017**	−32.09 (−135.09, 70.91)	0.53	0.01 (−0.08, 0.10)	0.83
	* sex	-	-	-	-	−0.03 (−0.21, 0.16)	0.79	-	-	-	-
	* diabetes status	-	-	-	-	0.04 (−0.10, 0.18)	0.55	-	-	-	-
LDL cholesterol (mmol/L)	Unadjusted	14.21 (−13.00, 41.42)	0.30	12.64 (−1.67, 26.95)	0.082	0.01 (−0.01, 0.03)	0.15	5.77 (−26.34, 37.89)	0.72	−0.003 (−0.03, 0.02)	0.84
	+age	9.02 (−17.71, 35.75)	0.50	13.08 (−1.65, 27.81)	0.080	0.01 (−0.01, 0.03)	0.23	−1.08 (−32.35, 30.19)	0.95	0.002 (−0.02, 0.03)	0.89
	+BMI	9.44 (−17.51, 36.39)	0.48	13.13 (−1.80, 28.05)	0.083	0.01 (−0.01, 0.03)	0.23	−1.03 (−32.71, 30.65)	0.95	0.002 (−0.03, 0.03)	0.90
	+education	10.94 (−15.87, 37.74)	0.42	14.31 (−0.19, 28.81)	0.053	0.01 (−0.01, 0.03)	0.22	−4.09 (−34.49, 26.31)	0.79	0.002 (−0.03, 0.03)	0.91
LDL:HDL ratio	Unadjusted	2.49 (−28.35, 33.34)	0.87	3.84 (−12.71, 20.40)	0.64	−0.001 (−0.02, 0.02)	0.92	4.69 (−31.21, 40.60)	0.79	−0.01 (−0.04, 0.02)	0.69
	+age	3.54 (−26.01, 33.09)	0.81	3.85 (−12.91, 20.62)	0.65	−0.001 (−0.02, 0.02)	0.96	6.14 (−28.09, 40.38)	0.72	−0.01 (−0.04, 0.02)	0.64
	+BMI	2.80 (−27.09, 32.69)	0.85	3.85 (−13.18, 20.88)	0.65	−0.001 (−0.02, 0.02)	0.94	5.95 (−28.89, 40.78)	0.73	−0.01 (−0.04, 0.02)	0.68
	+education	5.11 (−24.77, 34.99)	0.73	5.64 (−11.07, 22.36)	0.50	−0.0003 (−0.02, 0.02)	0.98	1.27 (−32.37, 34.92)	0.94	−0.01 (−0.04, 0.02)	0.67
Triglycerides (mmol/L)	Unadjusted	−23.01 (−46.28, 0.26)	0.052	−3.33 (−15.55, 8.90)	0.59	−0.02 (−0.04, −0.01)	**0.008**	22.17 (−4.23, 48.56)	0.10	−0.002 (−0.03, 0.02)	0.84
	+age	−19.95 (−42.54, 2.64)	0.082	−3.49 (−15.96, 8.97)	0.58	−0.02 (−0.04, −0.01)	**0.012**	26.71 (1.81, 51.60)	**0.036**	−0.01 (−0.03, 0.02)	0.67
	+BMI	−19.59 (−42.35, 3.17)	0.090	−3.48 (−16.11, 9.14)	0.58	−0.02 (−0.04, −0.004)	**0.014**	27.19 (1.86, 52.52)	**0.036**	−0.01 (−0.03, 0.02)	0.63
	+education	−19.55 (−42.75, 3.65)	0.097	−2.63 (−15.32, 10.05)	0.68	−0.02 (−0.04, −0.004)	**0.016**	23.88 (−0.75, 48.50)	0.057	−0.01 (−0.03, 0.02)	0.62
	* sex	-	-	-	-	−0.01 (−0.05, 0.03)	0.60	22.53 (−37.40, 82.46)	0.45	-	-
	* diabetes status	-	-	-	-	−0.02 (−0.06, 0.02)	0.25	74.10 (24.55, 123.66)	**0.004**	-	-

Data are reported as beta-coefficients (β) with 95% confidence intervals and corresponding *p*-values, derived from simple or multivariable general linear regression models. Bold denotes statistical significance at *p* < 0.05. * indicates interaction test effects, whereby only those outcomes with statistical significance in one or more models were tested for interactions with sex or diabetes status. ⊕ = higher scores indicate better cognitive performance; ⊖ = lower scores indicate better cognitive performance. Other CANTAB domains assessed are reported in Supplemental Table S1. Abbreviations: BMI, body mass index; HDL/LDL, high/low-density lipoprotein cholesterol; RTI, reaction time index; RVP, rapid visual processing.

**Table 4:** Univariable and multivariable associations between cardiometabolic variables and Trail Making Tests.

Predictor	Model	TMT-A (⊖)	*p*	TMT-B (⊖)	*p*	TMT B/A Ratio (⊖)	*p*
		β (95% CI)		β (95% CI)		β (95% CI)	
Body mass index (kg/m^2^)	Unadjusted	−0.24 (−0.62, 0.14)	0.21	−1.92 (−3.98, 0.14)	0.07	−0.05 (−0.16, 0.05)	0.30
	+age	−0.17 (−0.55, 0.22)	0.39	−1.75 (−3.89, 0.38)	0.11	−0.06 (−0.17, 0.05)	0.28
	+education	−0.24 (−0.65, 0.18)	0.26	−2.51 (−4.76, −0.25)	**0.03**	−0.09 (−0.21, 0.02)	0.11
	* sex	-	-	−1.87 (−6.31, 2.57)	0.40	-	-
	* diabetes status	-	-	−3.74 (−7.97, 0.48)	0.081	-	-
Waist-to-hip ratio	Unadjusted	6.34 (−11.27, 23.95)	0.47	10.66 (−91.30, 112.62)	0.83	−0.72 (−5.73, 4.29)	0.77
	+age	3.49 (−14.27, 21.25)	0.69	0.09 (−104.42, 104.60)	0.99	−0.72 (−5.06, 4.47)	0.78
	+education	2.64 (−15.65, 20.94)	0.72	−9.72 (−116.51, 97.08)	0.86	−1.24 (−6.53, 4.05)	0.64
Body fat (%)	Unadjusted	−0.20 (−0.42, 0.02)	0.069	−0.84 (−2.09, 0.40)	0.18	−0.01 (−0.07, 0.06)	0.86
	+age	−0.18 (−0.40, 0.04)	0.10	−0.77 (−2.03, 0.49)	0.22	−0.01 (−0.07, 0.06)	0.84
	+education	−0.18 (−0.40, 0.04)	0.11	−0.74 (−2.00, 0.52)	0.24	−0.01 (−0.07, 0.06)	0.88
Fasting glucose (mmol/L)	Unadjusted	0.14 (−1.12, 1.40)	0.83	−0.39 (−7.39, 6.62)	0.91	−0.06 (−0.40, 0.29)	0.74
	+age	−0.004 (−1.24, 1.23)	0.99	−0.87 (−7.93, 6.19)	0.81	−0.06 (−0.41, 0.29)	0.75
	+BMI	0.05 (−1.20, 1.29)	0.94	−0.34 (−7.31, 6.62)	0.92	−0.04 (−0.39, 0.31)	0.83
	+education	−0.04 (−1.30, 1.23)	0.95	−1.26 (−8.13, 5.62)	0.71	−0.08 (−0.43, 0.27)	0.65
2–h post-load glucose (mmol/L)	Unadjusted	0.02 (−0.47, 0.51)	0.94	−0.35 (−3.06, 2.36)	0.80	−0.03 (−0.16, 0.10)	0.67
	+age	−0.05 (−0.53, 0.43)	0.84	−0.59 (−3.33, 2.15)	0.67	−0.03 (−0.16, 0.11)	0.69
	+BMI	−0.11 (−0.61, 0.39)	0.67	−1.20 (−3.96, 1.56)	0.39	−0.05 (−0.19, 0.09)	0.49
	+education	−0.09 (−0.59, 0.42)	0.73	−0.96 (−3.68, 1.75)	0.48	−0.04 (−0.18, 0.10)	0.59
HbA1c (%)	Unadjusted	0.97 (−0.95, 2.90)	0.31	5.55 (−5.15, 16.24)	0.30	0.12 (−0.41, 0.65)	0.65
	+age	0.77 (−1.12, 2.67)	0.42	4.93 (−5.87, 15.73)	0.36	0.12 (−0.41, 0.66)	0.64
	+BMI	0.82 (−1.08, 2.73)	0.39	5.44 (−5.15, 16.04)	0.31	0.14 (−0.40, 0.68)	0.60
	+education	0.75 (−1.18, 2.67)	0.44	4.59 (−5.84, 15.02)	0.38	0.10 (−0.43, 0.64)	0.70
	* sex	3.89 (−1.65, 9.44)	0.16	−6.62 (−37.42, 24.18)	0.67	−1.09 (−2.64, 0.46)	0.16
	* diabetes status	0.73 (−4.31, 5.77)	0.77	0.28 (−27.15, 27.72)	0.98	−0.22 (−1.62, 1.18)	0.75
Total cholesterol (mmol/L)	Unadjusted	−0.92 (−2.50, 0.66)	0.25	−1.91 (−10.83, 7.00)	0.67	0.10 (−0.34, 0.54)	0.64
	+age	−1.17 (−2.72, 0.38)	0.13	−2.70 (−11.71, 6.30)	0.55	0.11 (−0.34, 0.55)	0.63
	+BMI	−1.21 (−2.76, 0.34)	0.12	−3.09 (−11.93, 5.76)	0.49	0.10 (−0.35, 0.54)	0.67
	+education	−1.32 (−2.88, 0.24)	0.094	−4.15 (−12.82, 4.52)	0.34	0.05 (−0.39, 0.50)	0.81
HDL cholesterol (mmol/L)	Unadjusted	−0.99 (−7.17, 5.19)	0.75	−3.00 (−37.32, 31.31)	0.86	0.02 (−1.66, 1.69)	0.99
	+age	−2.27 (−8.42, 3.88)	0.46	−7.10 (−42.22, 28.08)	0.69	0.05 (−1.69, 1.79)	0.96
	+BMI	−2.82 (−9.07, 3.43)	0.37	−12.51 (−47.35, 22.34)	0.47	−0.13 (−1.89, 1.63)	0.88
	+education	−2.81 (−9.06, 3.45)	0.37	−12.40 (−46.60, 21.80)	0.47	−0.12 (−1.87, 1.62)	0.89
LDL cholesterol (mmol/L)	Unadjusted	−1.11 (−2.88, 0.66)	0.21	−7.06 (−17.03, 2.90)	0.16	−0.13 (−0.62, 0.37)	0.60
	+age	−1.38 (−3.13, 0.38)	0.12	−8.13 (−18.21, 1.96)	0.11	−0.14 (−0.65, 0.37)	0.59
	+BMI	−1.41 (−3.17, 0.35)	0.11	−8.41 (−18.26, 1.43)	0.092	−0.15 (−0.65, 0.36)	0.57
	+education	−1.45 (−3.20, 0.31)	0.10	−8.76 (−18.38, 0.86)	0.073	−0.16 (−0.66, 0.34)	0.53
LDL:HDL ratio	Unadjusted	−1.34 (−3.29, 0.61)	0.17	−7.15 (−18.25, 3.94)	0.20	−0.06 (−0.61, 0.49)	0.84
	+age	−1.26 (−3.20, 0.68)	0.20	−6.87 (−18.04, 4.30)	0.22	−0.06 (−0.61, 0.50)	0.84
	+BMI	−1.17 (−3.13, 0.80)	0.24	−5.88 (−16.95, 5.18)	0.29	−0.02 (−0.58, 0.54)	0.94
	+education	−1.27 (−3.23, 0.69)	0.20	−6.75 (−17.60, 4.10)	0.22	−0.06 (−0.62, 0.50)	0.84
Triglycerides (mmol/L)	Unadjusted	−0.28 (−1.85, 1.29)	0.72	6.42 (−2.12, 14.96)	0.14	0.42 (0.01, 0.83)	**0.045**
	+age	0.04 (−1.54, 1.62)	0.96	7.92 (−0.76, 16.59)	0.073	0.44 (0.01, 0.86)	**0.045**
	+BMI	0.05 (−1.53, 1.64)	0.95	7.99 (−0.51, 16.49)	0.065	0.44 (0.01, 0.86)	**0.043**
	+education	0.01 (−1.58, 1.60)	0.99	7.56 (−0.77, 15.89)	0.074	0.42 (0.00, 0.84)	0.050
	* sex	-	-	-	-	0.09 (−0.94, 1.12)	0.86
	* diabetes status	-	-	-	-	−0.37 (−1.28, 0.55)	0.42

Data are reported as beta-coefficients (β) with 95% confidence intervals and corresponding *p*-values, derived from simple or multivariable general linear regression models. Bold denotes statistical significance at *p* < 0.05. * indicates interaction test effects, whereby only those outcomes with statistical significance in one or more models were tested for interactions with sex or diabetes status. ⊖ = lower scores indicate better cognitive performance. Other CANTAB domains assessed are reported in Supplemental Table S1. Abbreviations: BMI, body mass index; HDL/LDL, high/low-density lipoprotein cholesterol.; TMT, Trail Making Test.

**Table 5:** Univariable and multivariable associations between cardiometabolic variables with Stroop and Digit Symbol Substitution Test outcomes.

Predictor	Model	DSST Score (⊕)		Stroop Offtime (⊖)		Stroop Ontime (⊖)	
		β (95% CI)	*p*	β (95% CI)	*p*	β (95% CI)	*p*
Body mass index (kg/m^2^)	Unadjusted	0.53 (−0.57, 1.63)	0.34	−0.40 (−1.64, 0.84)	0.52	−0.46 (−1.51, 0.60)	0.39
	+age	0.18 (−0.89, 1.24)	0.74	−0.32 (−1.61, 0.97)	0.62	−0.01 (−0.96, 0.94)	0.99
	+education	0.30 (−0.85, 1.44)	0.61	0.37 (−0.92, 1.66)	0.56	−0.08 (−1.12, 0.96)	0.87
Waist-to-hip ratio	Unadjusted	−20.21 (−71.88, 31.46)	0.44	−5.39 (−58.52, 47.74)	0.84	23.89 (−25.76, 73.55)	0.34
	+age	−5.75 (−54.82, 43.32)	0.81	−11.66 (−65.95, 42.62)	0.67	5.97 (−38.28, 50.22)	0.26
	+education	−4.48 (−54.96, 46.00)	0.86	6.07 (−42.21, 54.36)	0.81	5.06 (−40.62, 50.74)	0.82
Body fat (%)	Unadjusted	0.49 (−0.16, 1.13)	0.14	−0.42 (−1.15, 0.30)	0.25	−0.25 (−0.88, 0.38)	0.43
	+age	0.39 (−0.22, 0.99)	0.20	−0.40 (−1.14, 0.34)	0.28	−0.11 (−0.66, 0.44)	0.69
	+education	0.40 (−0.22, 1.01)	0.20	−0.45 (−1.13, 0.24)	0.20	−0.10 (−0.66, 0.45)	0.71
Fasting glucose (mmol/L)	Unadjusted	−0.80 (−4.43, 2.83)	0.66	−0.57 (−4.65, 3.51)	0.78	1.32 (−2.16, 4.79)	0.45
	+age	−0.16 (−3.56, 3.24)	0.93	−0.76 (−4.90, 3.37)	0.71	0.54 (−2.50, 3.59)	0.72
	+BMI	−0.21 (−3.67, 3.24)	0.90	−0.67 (−4.87, 3.52)	0.75	0.55 (−2.54, 3.65)	0.72
	+education	−0.02 (−3.57, 3.52)	0.99	0.14 (−3.80, 4.08)	0.94	0.48 (−2.69, 3.64)	0.76
Two-hour post-load glucose (mmol/L)	Unadjusted	0.12 (−1.33, 1.57)	0.86	0.65 (−0.92, 2.22)	0.41	0.12 (−1.23, 1.48)	0.85
	+age	0.48 (−0.87, 1.84)	0.48	0.58 (−1.02, 2.18)	0.47	−0.26 (−1.44, 0.93)	0.67
	+BMI	0.57 (−0.84, 1.98)	0.42	0.51 (−1.16, 2.18)	0.54	−0.27 (−1.51, 0.96)	0.66
	+education	0.59 (−0.83, 2.01)	0.41	0.29 (−1.27, 1.86)	0.71	−0.25 (−1.51, 1.00)	0.69
HbA1c (%)	Unadjusted	−1.94 (−7.54, 3.67)	0.49	0.18 (−6.12, 6.49)	0.95	1.83 (−3.55, 7.20)	0.50
	+age	−0.98 (−6.24, 4.27)	0.71	−0.09 (−6.48, 6.31)	0.98	0.68 (−4.02, 5.39)	0.77
	+BMI	−1.04 (−6.36, 4.28)	0.70	0.004 (−6.46, 6.47)	1.00	0.69 (−4.08, 5.46)	0.77
	+education	−0.81 (−6.25, 4.63)	0.76	0.83 (−5.19, 6.86)	0.78	0.61 (−4.24, 5.45)	0.80
Total cholesterol (mmol/L)	Unadjusted	−0.65 (−5.32, 4.02)	0.78	0.42 (−4.78, 5.63)	0.87	−0.18 (−4.64, 4.28)	0.93
	+age	0.32 (−4.06, 4.70)	0.88	0.15 (−5.16, 5.45)	0.96	−1.42 (−5.30, 2.46)	0.46
	+BMI	0.37 (−4.07, 4.80)	0.87	0.08 (−5.28, 5.44)	0.98	−1.43 (−5.36, 2.50)	0.47
	+education	0.52 (−3.98, 5.03)	0.82	1.01 (−4.00, 6.02)	0.69	−1.55 (−5.56, 2.45)	0.44
HDL cholesterol (mmol/L)	Unadjusted	7.61 (−9.89, 25.11)	0.39	−12.44 (−31.22, 6.35)	0.19	−1.19 (−18.38, 16.00)	0.89
	+age	13.44 (−2.81, 29.70)	0.10	−14.80 (−34.00, 4.39)	0.13	−7.98 (−23.02, 7.05)	0.29
	+BMI	14.38 (−2.25, 31.02)	0.088	−15.94 (−35.59, 3.70)	0.11	−8.25 (−23.71, 7.21)	0.29
	+education	14.50 (−2.26, 31.26)	0.088	−16.03 (−34.80, 2.75)	0.092	−8.23 (−23.84, 7.38)	0.29
LDL cholesterol (mmol/L)	Unadjusted	0.82 (−4.35, 6.00)	0.75	2.14 (−3.52, 7.79)	0.45	−1.29 (−6.28, 3.70)	0.61
	+age	2.06 (−2.83, 6.95)	0.40	1.85 (−3.95, 7.66)	0.52	−2.81 (−7.22, 1.60)	0.21
	+BMI	2.09 (−2.86, 7.04)	0.40	1.82 (−4.05, 7.69)	0.54	−2.82 (−7.29, 1.65)	0.21
	+education	2.22 (−2.78, 7.23)	0.37	2.07 (−3.56, 7.70)	0.46	−2.87 (−7.39, 1.64)	0.21
LDL:HDL ratio	Unadjusted	0.42 (−5.34, 6.19)	0.88	4.63 (−1.53, 10.78)	0.14	−2.27 (−7.78, 3.25)	0.41
	+age	−0.01 (−5.43, 5.40)	0.99	4.78 (−1.42, 10.98)	0.13	−1.72 (−6.61, 3.16)	0.48
	+BMI	−0.12 (−5.64, 5.39)	0.96	4.99 (−1.31, 11.28)	0.12	−1.74 (−6.72, 3.25)	0.49
	+education	0.05 (−5.54, 5.64)	0.99	5.64 (−0.35, 11.63)	0.064	−1.86 (−6.92, 3.19)	0.46
Triglycerides (mmol/L)	Unadjusted	−3.02 (−7.49, 1.45)	0.18	0.61 (−4.49, 5.71)	0.81	0.98 (−3.38, 5.34)	0.65
	+age	−4.68 (−8.78, −0.58)	**0.026**	1.07 (−4.20, 6.35)	0.68	2.91 (−0.87, 6.70)	0.13
	+BMI	−4.69 (−8.83, −0.55)	**0.027**	1.09 (−4.23, 6.41)	0.68	2.91 (−0.92, 6.75)	0.13
	+education	−4.61 (−8.81, −0.40)	**0.033**	1.51 (−3.42, 6.45)	0.54	2.88 (−1.00, 6.76)	0.14
	* sex	−0.59 (−10.38, 9.20)	0.90	-	-	-	-
	* diabetes status	−4.57 (−13.69, 4.55)	0.32	-	-	-	-

Data are reported as beta-coefficients (β) with 95% confidence intervals and corresponding *p*-values, derived from simple or multivariable general linear regression models. Bold denotes statistical significance at *p* < 0.05. * indicates interaction test effects, whereby only those outcomes with statistical significance in one or more models were tested for interactions with sex or diabetes status. ⊕ = higher scores indicate better cognitive performance; ⊖ = lower scores indicate better cognitive performance. Other CANTAB domains assessed are reported in Supplemental Table S1. Abbreviations: BMI, body mass index; DSST, Digit Symbol Substitution Test; HDL/LDL, high/low-density lipoprotein cholesterol.

**Table 6:** Standardised effect magnitudes for significant cardiometabolic–cognitive associations.

Association	Fully Adjusted β (95% CI)	*p*	Per 1 SD of Predictor
Triglycerides → DSST	−4.61 (−8.81, −0.40)	0.033	−0.32 SD
BMI → TMT-B	−2.51 (−4.76, −0.25)	0.03	−0.35 SD
HDL-C → RVP A-prime	0.08 (0.02, 0.15)	0.017	+0.35 SD
Triglycerides → RVP A-prime	−0.02 (−0.04, −0.004)	0.016	−0.36 SD
HbA1c → RVP Probability of False Alarms	0.04 (0.01, 0.07)	0.015	+0.41 SD

Standardised estimates were derived as β × SD (predictor)/SD (outcome), expressing associations per one standard deviation of the cardiometabolic exposure in standard deviation units of the cognitive outcome. Models were adjusted for age, BMI, and level of education (except the association between BMI and TMT-B; where the model was adjusted for age and level of education). Abbreviations: BMI, body mass index; DSST, Digit Symbol Substitution Test; HDL/LDL, high/low-density lipoprotein cholesterol; RVP, Rapid Visual Processing; TMT, Trail Making Test.

## Data Availability

Restrictions apply to the availability of some, or all, data generated or analysed during this study to preserve patient confidentiality, or because they were used under licence. The corresponding author will, upon request, detail the restrictions and any conditions under which access to some data may be provided.

## Supplementary Materials

The following supporting information can be downloaded at: https://www.mdpi.com/article/10.3390/medsci14050520/s1, Table S1: Univariable and multivariable associations between cardiometabolic variables and selected CANTAB measures. Figure S1: Participant timeline: Schedule of enrolment, interventions, and assessments. STROBE Statement—Checklist of items that should be included in reports of cross-sectional studies.
