# Supplementary material for: Cardiometabolic Risk Factors and Domain-Specific Cognitive Function in Prediabetes and Type 2 Diabetes
Source: Med Sci (Basel). 2026 Aug 26;14(5):520. doi: 10.3390/medsci14050520 (PMC13609006; doi:10.3390/medsci14050520)
Supplement: Supplementary file 1 [file medsci-14-00520-s001.zip › medsci-4499725-supplementary.pdf]

Supplemental Table S1. Univariable and multivariable associations between cardiometabolic variables and selected CANTAB measures

Predictor	Model	DMS percent correct (Simultaneous) (⊕) β (95% CI)	*P*	DMS percent correct (all delays) (⊕) β (95% CI)	*P*	Motor organisation task median latency (⊖) β (95% CI)	*P*	PAL total errors adjusted (⊖) β (95% CI)	*P*	Spatial working memory between errors (⊖) β (95% CI)	*P*	Spatial working memory strategy (⊖) β (95% CI)	*P*
Body mass index (kg/m^2)	*Unadjusted*	-0.07 (-0.78, 0.63)	0.84	0.47 (-0.41, 1.35)	0.28	-5.63 (-19.69, 8.43)	0.42	-0.44 (-1.45, 0.58)	0.39	-0.33 (-1.01, 0.35)	0.34	-0.03 (-0.25, 0.20)	0.81
	+ *age*	-0.10 (-0.83, 0.63)	0.79	0.43 (-0.49, 1.34)	0.36	-4.14 (-18.59, 10.31)	0.57	-0.30 (-1.33, 0.73)	0.56	-0.38 (-1.09, 0.34)	0.30	-0.03 (-0.26, 0.21)	0.83
	+ *education*	-0.01 (-0.78, 0.77)	0.99	0.52 (-0.45, 1.50)	0.29	-6.14 (-21.58, 9.29)	0.43	-0.37 (-1.48, 0.73)	0.50	-0.44 (-1.21, 0.32)	0.25	-0.04 (-0.30, 0.21)	0.73
Waist-to-Hip ratio	*Unadjusted*	-9.13 (-39.83, 21.58)	0.55	-20.31 (-58.71, 18.09)	0.32	129.79 (-489.76, 749.34)	0.68	0.28 (-46.11, 46.67)	0.99	-14.53 (-44.84, 15.79)	0.34	-1.70 (-11.61, 8.21)	0.73
	+ *age*	-8.78 (-40.55, 22.98)	0.58	-18.49 (-58.11, 21.14)	0.38	62.92 (-571.17, 697.02)	0.84	-9.27 (-57.11, 38.57)	0.7	-14.32 (-45.61, 16.98)	0.36	-1.91 (-12.14, 8.32)	0.71
	+ *education*	-7.08 (-39.42, 25.27)	0.66	-18.12 (-58.72, 22.47)	0.37	36.96 (-614.30, 688.22)	0.91	-9.82 (-58.73, 39.09)	0.69	-15.10 (-47.25, 17.06)	0.35	-2.27 (-12.76, 8.23)	0.67
Body fat (%)	*Unadjusted*	0.16 (-0.22, 0.54)	0.41	0.20 (-0.28, 0.68)	0.41	-1.38 (-9.15, 6.39)	0.72	0.05 (-0.51, 0.62)	0.85	0.21 (-0.17, 0.58)	0.28	0.05 (-0.07, 0.18)	0.40
	+ *age*	0.15 (-0.24, 0.54)	0.43	0.18 (-0.31, 0.67)	0.47	-0.83 (-8.66, 7.01)	0.83	0.13 (-0.44, 0.70)	0.64	0.20 (-0.18, 0.59)	0.30	0.05 (-0.07, 0.18)	0.39
	+ *education*	0.16 (-0.23, 0.55)	0.42	0.18 (-0.32, 0.68)	0.46	-0.76 (-8.67, 7.15)	0.85	0.13 (-0.44, 0.71)	0.65	0.20 (-0.19, 0.59)	0.30	0.05 (-0.07, 0.18)	0.40
Fasting glucose (mmol/L)	*Unadjusted*	1.26 (-1.17, 3.69)	0.30	-0.18 (-3.29, 2.94)	0.91	8.42 (-34.72, 51.56)	0.70	-1.51 (-4.58, 1.56)	0.33	-0.61 (-2.72, 1.50)	0.56	-0.38 (-1.06, 0.30)	0.27
	+ *age*	1.34 (-1.15, 3.83)	0.28	-0.004 (-3.19, 3.18)	1.00	5.23 (-38.33, 48.79)	0.81	-1.75 (-4.79, 1.29)	0.25	-0.58 (-2.74, 1.57)	0.59	-0.39 (-1.08, 0.30)	0.26
	+ *BMI*	1.33 (-1.20, 3.85)	0.29	0.07 (-3.13, 3.27)	0.97	6.13 (-37.89, 50.15)	0.78	-1.70 (-4.78, 1.38)	0.27	-0.52 (-2.67, 1.64)	0.63	-0.39 (-1.09, 0.31)	0.27
	+ *education*	1.52 (-1.04, 4.07)	0.24	0.23 (-3.03, 3.49)	0.89	3.57 (-41.28, 48.41)	0.87	-1.86 (-5.01, 1.29)	0.24	-0.64 (-2.86, 1.57)	0.56	-0.43 (-1.15, 0.28)	0.23
Two-hour post-load glucose (mmol/L)	*Unadjusted*	0.63 (-0.18, 1.45)	0.12	0.11 (-0.94, 1.17)	0.83	-1.69 (-18.03, 14.65)	0.84	-0.54 (-1.79, 0.71)	0.39	-0.33 (-1.16, 0.49)	0.42	-0.15 (-0.41, 0.12)	0.27
	+ *age*	0.68 (-0.16, 1.51)	0.11	0.19 (-0.90, 1.27)	0.73	-3.48 (-20.07, 13.11)	0.68	-0.63 (-1.87, 0.61)	0.31	-0.32 (-1.17, 0.52)	0.45	-0.16 (-0.43, 0.12)	0.25
	+ *BMI*	0.70 (-0.18, 1.58)	0.12	0.35 (-0.77, 1.48)	0.53	-4.85 (-22.02, 12.32)	0.57	-0.76 (-2.05, 0.52)	0.24	-0.46 (-1.33, 0.41)	0.29	-0.17 (-0.46, 0.11)	0.22
	+ *education*	0.72 (-0.17, 1.60)	0.11	0.37 (-0.77, 1.51)	0.51	-4.65 (-21.92, 12.63)	0.59	-0.76 (-2.06, 0.54)	0.24	-0.48 (-1.36, 0.40)	0.28	-0.18 (-0.47, 0.11)	0.21
HbA1c (%)	*Unadjusted*	1.87 (-1.71, 5.46)	0.30	-0.81 (-5.41, 3.79)	0.72	44.56 (-21.56, 110.69)	0.18	-2.14 (-6.91, 2.63)	0.37	0.23 (-3.08, 3.53)	0.89	-0.03 (-1.10, 1.04)	0.96
	+ *age*	1.99 (-1.67, 5.66)	0.28	-0.58 (-5.27, 4.12)	0.81	40.32 (-26.58, 107.21)	0.23	-2.62 (-7.36, 2.12)	0.27	0.28 (-3.08, 3.65)	0.87	-0.04 (-1.13, 1.06)	0.94
	+ *BMI*	1.96 (-1.77, 5.70)	0.30	-0.36 (-5.09, 4.37)	0.88	40.24 (-27.21, 107.68)	0.24	-2.64 (-7.42, 2.14)	0.27	0.28 (-3.09, 3.64)	0.87	-0.04 (-1.15, 1.07)	0.94
	+ *education*	2.17 (-1.60, 5.94)	0.25	-0.18 (-4.99, 4.63)	0.94	37.79 (-30.57, 106.16)	0.27	-2.77 (-7.62, 2.08)	0.26	0.15 (-3.30, 3.59)	0.93	-0.08 (-1.21, 1.05)	0.89
Total cholesterol (mmol/L)	*Unadjusted*	-1.32 (-3.93, 1.29)	0.31	0.96 (-2.37, 4.29)	0.56	11.88 (-41.75, 65.51)	0.66	0.16 (-3.92, 4.23)	0.94	-0.80 (-3.44, 1.84)	0.55	-0.07 (-0.93, 0.79)	0.88
	+ *age*	-1.31 (-4.00, 1.38)	0.33	1.24 (-2.18, 4.65)	0.47	6.39 (-48.32, 61.11)	0.82	-0.30 (-4.39, 3.78)	0.88	-0.76 (-3.49, 1.96)	0.58	-0.08 (-0.97, 0.80)	0.85
	+ *BMI*	-1.36 (-4.09, 1.38)	0.32	1.40 (-2.03, 4.83)	0.42	5.15 (-50.20, 60.51)	0.85	-0.38 (-4.51, 3.75)	0.86	-0.89 (-3.62, 1.84)	0.51	-0.09 (-0.99, 0.81)	0.84
	+ *education*	-1.24 (-4.02, 1.55)	0.37	1.57 (-1.91, 5.06)	0.37	1.29 (-55.37, 57.95)	0.96	-0.60 (-4.89, 3.69)	0.78	-1.01 (-3.79, 1.77)	0.47	-0.12 (-1.04, 0.80)	0.79
HDL cholesterol (mmol/L)	*Unadjusted*	-5.42 (-15.51, 4.66)	0.28	2.27 (-10.67, 15.22)	0.73	73.05 (-121.95, 268.04)	0.45	-4.00 (-20.05, 12.06)	0.62	-5.00 (-15.14, 5.15)	0.33	-1.58 (-4.87, 1.72)	0.34
	+ *age*	-5.40 (-15.82, 5.02)	0.30	3.27 (-10.02, 16.55)	0.62	55.24 (-143.90, 254.38)	0.58	-5.54 (-21.51, 10.43)	0.49	-4.93 (-15.41, 5.55)	0.35	-1.69 (-5.09, 1.71)	0.32
	+ *BMI*	-5.71 (-16.34, 4.93)	0.29	4.20 (-9.22, 17.62)	0.53	44.38 (-158.28, 247.04)	0.66	-6.16 (-22.39, 10.07)	0.45	-5.85 (-16.39, 4.70)	0.27	-1.78 (-5.26, 1.69)	0.31
	+ *education*	-5.67 (-16.37, 5.02)	0.29	4.23 (-9.30, 17.76)	0.53	44.52 (-160.60, 249.63)	0.66	-6.47 (-22.81, 9.87)	0.43	-5.86 (-16.51, 4.78)	0.27	-1.79 (-5.30, 1.72)	0.31

LDL cholesterol (mmol/L)	*Unadjusted*	-0.69 (-3.70, 2.32)	0.65	1.85 (-1.94, 5.64)	0.33	-3.71 (-60.08, 52.66)	0.90	-0.73 (-5.47, 4.01)	0.76	-1.11 (-4.05, 1.82)	0.45	-0.15 (-1.09, 0.79)	0.75
	+ age	-0.68 (-3.79, 2.43)	0.66	2.17 (-1.71, 6.05)	0.27	-7.35 (-65.14, 50.44)	0.80	-1.22 (-5.98, 3.55)	0.61	-0.93 (-3.95, 2.09)	0.54	-0.11 (-1.09, 0.86)	0.82
	+ BMI	-0.69 (-3.84, 2.46)	0.66	2.21 (-1.68, 6.10)	0.26	-8.59 (-66.60, 49.42)	0.77	-1.25 (-6.06, 3.57)	0.60	-1.00 (-4.01, 2.00)	0.50	-0.12 (-1.11, 0.86)	0.81
	+ education	-0.59 (-3.77, 2.60)	0.71	2.34 (-1.59, 6.27)	0.24	-8.24 (-67.19, 50.70)	0.78	-1.50 (-6.37, 3.38)	0.54	-1.08 (-4.12, 1.96)	0.48	-0.14 (-1.14, 0.86)	0.78
LDL: HDL ratio	*Unadjusted*	0.88 (-2.48, 4.24)	0.60	1.03 (-3.25, 5.30)	0.63	-34.60 (-96.87, 27.66)	0.27	-0.49 (-5.50, 4.52)	0.84	-0.35 (-3.66, 2.96)	0.83	0.01 (-1.05, 1.07)	0.98
	+ age	0.87 (-2.53, 4.28)	0.61	0.98 (-3.33, 5.29)	0.65	-34.00 (-96.78, 28.79)	0.28	-0.42 (-5.41, 4.56)	0.86	-0.39 (-3.72, 2.94)	0.81	0.002 (-1.07, 1.07)	1.00
	+ BMI	0.92 (-2.54, 4.39)	0.60	0.80 (-3.55, 5.14)	0.71	-31.89 (-95.16, 31.39)	0.32	-0.32 (-5.37, 4.73)	0.90	-0.23 (-3.56, 3.09)	0.89	0.02 (-1.07, 1.10)	0.97
	+ education	1.11 (-2.40, 4.62)	0.53	0.99 (-3.43, 5.40)	0.65	-31.70 (-96.25, 32.85)	0.33	-0.56 (-5.68, 4.56)	0.83	-0.35 (-3.73, 3.03)	0.84	-0.01 (-1.12, 1.09)	0.98
Triglycerides (mmol/L)	*Unadjusted*	-0.64 (-3.19, 1.92)	0.62	-1.01 (-4.25, 2.22)	0.53	-19.70 (-70.42, 31.02)	0.44	1.93 (-1.71, 5.58)	0.29	0.29 (-2.20, 2.78)	0.82	0.11 (-0.70, 0.92)	0.79
	+ age	-0.69 (-3.30, 1.92)	0.60	-1.18 (-4.46, 2.10)	0.47	-16.48 (-67.63, 34.68)	0.52	2.22 (-1.39, 5.84)	0.22	0.25 (-2.30, 2.80)	0.84	0.12 (-0.71, 0.95)	0.77
	+ BMI	-0.74 (-3.39, 1.92)	0.58	-1.03 (-4.35, 2.28)	0.53	-17.18 (-68.78, 34.42)	0.51	2.16 (-1.50, 5.82)	0.24	0.18 (-2.37, 2.73)	0.89	0.11 (-0.72, 0.95)	0.79
	+ education	-0.61 (-3.31, 2.08)	0.65	-0.91 (-4.28, 2.46)	0.59	-19.81 (-71.98, 32.36)	0.45	2.11 (-1.62, 5.83)	0.26	0.08 (-2.53, 2.69)	0.95	0.09 (-0.77, 0.95)	0.84

Data are reported as beta-coefficients (β) with 95% confidence intervals and corresponding p-values, derived from simple (unadjusted) or multivariable general linear regression models.

⊕ = higher scores indicate better cognitive performance; ⊖ = lower scores indicate better cognitive performance.

Abbreviations: BMI, body mass index; HDL/LDL, high/low-density lipoprotein cholesterol.
