# Supplementary material for: Cardiometabolic Risk Factors and Domain-Specific Cognitive Function in Prediabetes and Type 2 Diabetes
Source: Med Sci (Basel). 2026 Aug 26;14(5):520. doi: 10.3390/medsci14050520 (PMC13609006; doi:10.3390/medsci14050520)
Supplement: Supplementary file 1 [file medsci-14-00520-s001.zip › SPIRIT figure.pdf]

Figure S1: Participant timeline: Schedule of enrolment, interventions, and assessments.

	TRIAL PERIOD						
	Enrollment			Post-randomization		Close-out	
TIMEPOINT	$t_{week\ -1}$	$t_{week\ 1}$ (Days 1-2)		$t_{week\ 1}$ (Days 3-7)	$t_{week\ 14}$	$t_{week\ 15}$ (Days 1-2)	
ENROLLMENT:							
Eligibility screen (Medical History Oral Glucose Tolerance test HbA1c)	X						
Informed consent	X						
Randomisation			X				
INTERVENTION/ COMPARATOR:							
Carnosine			X →	→	→		
Placebo			X →	→	→		
ASSESSMENTS: *BASELINE:* Cardiovascular assessment (Endopat, Compilor) Dual energy x-ray absorbtiometry (DEXA) Peripheral Quantitative Computed Tomography (pQCT) Blood tests (Full blood Examination, Urea Electrolytes and Creatinine, Inflammatory Markers) Urine collection Stool collection Buccal Cell Collection		X					
Neuropsychological tests (Cambridge			X				

Automated Neuropsychological Automated Test Battery, Stroop, Digit Symbol Substitution Test, Trail Making Tests A/B							
**Questionnaires** (3-day food diary, Depression questionnaire PHQ-8, EQ-5D, Knee & Back Pain, Physical Activity, Pittsburg Sleep Quality Index (PSQI), Sleep Diary, Standford Sleepiness Scale)			X				
**Liver Fibroscan**			X				
**Fitbit**		X					
FOLLOW UP: **Oral Glucose Tolerance test** **Cardiovascular assessment** (Endopat, Compilor) **Dual energy x-ray absorbtiometry** (DEXA) **Peripheral Quantitative Computed Tomography** (pQCT) **Blood tests** (Full blood Examination, Urea Electrolytes and Creatinine, Inflammatory Markers, HbA1c) **Urine collection** **Stool collection** **Buccal Cell Collection**						X	
**Neuropsychological tests** (Cambridge Automated Neuropsychological Automated Test Battery, Stroop, Digit							X

Symbol Substitution Test, Trail Making Tests A/B)							
**Questionnaires** (3-day food diary, Depression questionnaire PHQ-8, EQ-5D, Knee & Back Pain, Physical Activity, Pittsburg Sleep Quality Index (PSQI), Sleep Diary, Standford Sleepiness Scale)						X	
**Liver Fibroscan**							X
**Fitbit**						X	

Citation: Chan A-W, Boutron I, Hopewell S, Moher D, Schulz KF, et al. SPIRIT 2025 statement: updated guideline for protocols of randomised trials. BMJ 2025;389:e081477. https://dx.doi.org/10.1136/bmj-2024-081477

© 2025 Chan A-W et al. This is an Open Access article distributed under the terms of the Creative Commons Attribution License (https://creativecommons.org/licenses/by/4.0/), which permits unrestricted use, distribution, and reproduction in any medium, provided the original work is properly cited.
